# Comparative RNA sequencing reveals that HPV16 E6 abrogates the effect of E6*I on ROS metabolism

**DOI:** 10.1038/s41598-019-42393-6

**Published:** 2019-04-11

**Authors:** Philippe Paget-Bailly, Koceila Meznad, Diane Bruyère, Jérôme Perrard, Michael Herfs, Alain C. Jung, Christiane Mougin, Jean-Luc Prétet, Aurélie Baguet

**Affiliations:** 1 EA3181, LabEx LipSTIC ANR-11-LABX-0021, UFR Santé, 19 rue Ambroise Paré, Besançon, France; 2 Université Bourgogne Franche Comté, Besançon, France; 3 Centre Hospitalier Régional Universitaire, CNR HPV, 3 Bvd Alexandre Fleming, Besançon, France; 4 Laboratory of Experimental Pathology, GIGA-Cancer, University of Liege, Liege, Belgium; 5 Université de Strasbourg, Inserm, UMR_S1113, Centre de lutte contre le cancer Paul STRAUSS, Strasbourg, France

## Abstract

High-risk Human Papillomavirus infections are responsible for anogenital and oropharyngeal cancers. Alternative splicing is an important mechanism controlling HPV16 gene expression. Modulation in the splice pattern leads to polycistronic HPV16 early transcripts encoding a full length E6 oncoprotein or truncated E6 proteins, commonly named E6*. Spliced E6*I transcripts are the most abundant RNAs produced in HPV-related cancers. To date, the biological function of the E6*I isoform remains controversial. In this study, we identified, by RNA sequencing, cellular targets deregulated by E6*I, among which genes related to ROS metabolism. Concomitantly, E6*I-overexpressing cells display high levels of ROS. However, co-overexpression of both E6 and E6*I has no effect on ROS production. In HPV16-infected cells expressing different E6/E6*I levels, we show that the newly identified targets CCL2 and RAC2 are increased by E6*I but decreased by E6 expression, suggesting that E6 abrogates the effect of E6*I. Taken together, these data support the idea that E6*I acts independently of E6 to increase ROS production and that E6 has the ability to counteract the effects of E6*I. This asks the question of how E6*I can be considered separately of E6 in the natural history of HPV16 infection.

## Introduction

Human papillomaviruses (HPVs) are small non-enveloped viruses that present a tropism for squamous epithelium. More than 200 types of HPVs have been described to infect humans^1^. Based on their oncogenic potential, these viruses are classified in high-risk HPV (hrHPV), including HPV16 and HPV18, and low-risk HPV (lrHPV), including HPV6 and HPV11. HPV infections are responsible for cervical intraepithelial lesions that can progress to cancers, but they also cause a large fraction of anal, vulvar, vaginal, penile cancers, and a rising number of oropharyngeal cancers.

HPV16 is the most prevalent type in HPV-associated cancers^2^. Its genome contains a long control region (LCR), 6 open reading frames (ORFs) encoding early (E) proteins under the control of p97 promoter located in the LCR, and 2 ORFs encoding late (L) proteins under the control of p670 promoter located within the E7 ORF^3^. Viral proteins are produced through the translation of at least 20 polycistronic transcripts obtained by alternative splicing. At least 10 of these transcripts allow the production of the 2 major viral oncoproteins, E6 and E7, but also E6-truncated proteins, E6*I, E6*II and E6^E7^4,5^. hrHPV E6 and E7 proteins are consistently expressed in HPV-associated cancers^6,7^ and interact with many host cellular proteins. Notably, E6 and E7 proteins target p53 and pRB, respectively, for proteasome-mediated degradation, and thus inactivate these tumor suppressors^6,8,9^. More than 30 years ago, it was observed that the most abundant HPV16 transcript produced was spliced from the donor site 226 (SD 226) to the acceptor site 409 (SA409), both sites located in E6 ORF^10,11^. Interestingly, only hrHPVs harbor these splice sites, indicating that E6 ORF splicing events could be relevant for HPV-driven carcinogenesis^10^. Several studies and unpublished data from our laboratory also reported increased levels of spliced E6*I mRNA correlating with the severity of cervical lesions^5,12–14^. Concomitantly, it was proposed that the ratio of E6*I/E6*II spliced variants can be used as a predictive marker of clinical outcome in HPV-related cervical lesions^13^ and oropharyngeal cancers^15^.

Even if HPV16 early transcripts detection is used as a tool in screening and investigating HPV-related neoplasia, the biological significance of E6 splicing and ensuing E6*I protein expression remains elusive. It has been proposed that E6 ORF splicing facilitates translation re-initiation of the E7 ORF by increasing the space between E6 and E7 ORF^16,17^. However, other study showed that E7 is preferentially translated from the unspliced E6/E7 transcript rather than from the E6*I/E7 one, suggesting that this splicing event regulates E6 expression but not E7^18,19^. Apart from HPV gene expression regulation, the roles of E6*I ORF product in HPV life cycle and carcinogenesis also remains unclear, although a variety of functions have been reported for the truncated isoform. E6*I inhibits E6-mediated degradation of p53^20^, causes the degradation of some PDZ proteins, such as Dlg, PATJ and MAGI-1^21,22^, and modulates the expression of a subset of cellular factors involved in stress response, such as aldo-keto reductase genes^23^, superoxide dismutase isoform 2 (SOD2), and glutathione peroxidase 1 (GPX1), leading to the accumulation of reactive oxygen species (ROS)^24^. *In vivo* studies have shown that ectopic E6*I reduces tumor growth of both HPV-positive and HPV-negative cells in a xenograft nude mouse model^25^. Depending on the cellular context and especially the presence or absence of the E6 protein, E6*I seems implicated in different cellular pathways and its functions remain controversial^26^.

To get a better understanding on the underlying molecular mechanisms driving HPV-related carcinogenesis, the present study used RNA sequencing technology to analyze the impact of HPV16 E6*I isoform on cellular gene expression.

## Results

### HPV16 E6 and E6*I expression in U-2 OS HPV-negative cell line

**Figure 1:**
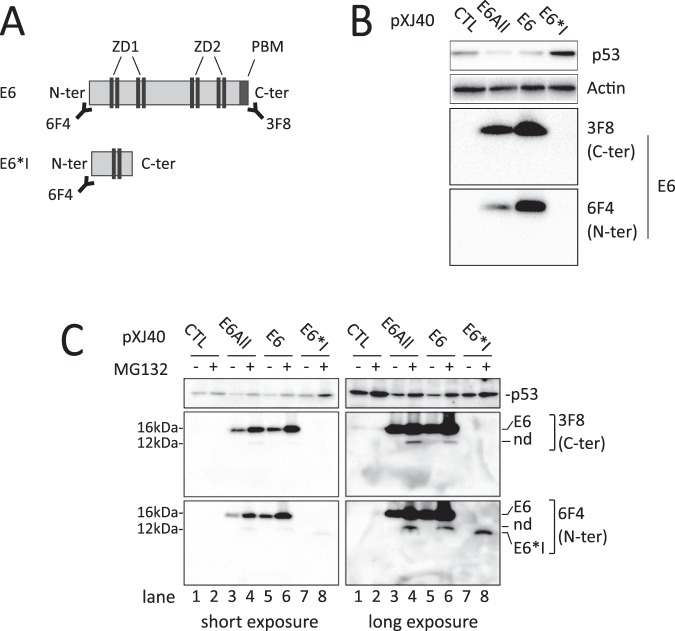
Transient transfection of E6 expression vectors in U-2 OS HPV-negative cell line. (**A)** Schematic representation of the E6 and E6*I protein isoforms with indicated regions recognized by the two E6-specific antibodies. The 6F4 antibody can recognize the N-terminal part of the E6 and E6*I proteins and the 3F8 antibody can target the C-terminal part of E6 protein. **(B)** Western blotting analysis showing p53, E6 and E6*I protein levels in U-2 OS cells transiently transfected with the pXJ40 empty vector (CTL), pXJ40-E6All vector (E6All), pXJ40-E6 vector (E6) or pXJ40-E6*I vector (E6*I). After 48-hour incubation, cells were treated either with DMSO or 10 μM MG132 proteasome inhibitor for 16 hours. The membrane was then incubated with the two E6 antibodies. For the detection of E6*I (corresponding to the first 43 amino acids of E6 protein), only the 6F4 antibody can be used. “nd” referred to a band at 11 kDa corresponding to a cleavage product observed in U-E6All cells treated with MG132. Full-length blots are presented in Figure S3.

HPV-negative U-2 OS cell line was transfected with expression vectors encoding either all HPV16 E6 isoforms (pXJ40-E6All or pXJ40bGlo∆int-E6All) or E6 (pXJ40-E6 or pXJ40bGlo∆int-E6) or E6*I (pXJ40-E6*I) exclusively or with empty vectors (pXJ40 or pXJ40-bGlo∆int) (Figs S1 and S2). We compared alternative splicing profiles of E6 transcripts obtained from these different E6-expression vectors. An accumulation of E6 unspliced transcripts produced from the pXJ40bGlo∆int-E6All vectors is observed, indicating that the presence of the β-globin intron in the pXJ40 vector favor the splicing between SD226 and SA409 splice sites, and consequently the production of the E6*I RNAs (Fig. S2). Western blot analysis were performed with two E6-specific antibodies. The 6F4 antibody recognizes the N-terminal part of the E6 protein, while the 3F8 antibody targets the C-terminal part of E6 (Fig. 1A). Consequently, the 3F8 antibody does not recognize the E6*I protein and the 6F4 antibody recognizes both E6 isoforms. The full length E6 protein was detected with both E6-directed antibodies in cells transiently expressing the pXJ40-E6All and pXJ40-E6 vectors. No E6*I protein was observed in pXJ40-E6All or pXJ40-E6*I expressing cells. But consistently with its role as a potent E6 antagonist, we observed an increase of p53 protein level in pXJ40-E6*I transfected cells. (Figs 1B and S3A).

MG-132 treatment, a proteasome inhibitor, was used on transfected cells to reveal a specific band with the 6F4 antibody that corresponds to the E6*I isoform (Fig. 1C, lane 8 and Fig. S3B). This indicate that an E6*I protein can be produced from the pXJ40-E6*I vector but also suggest that low levels of E6*I protein can be explain, at least in part, by its rapid turnover in these cells.

### Establishment of stable cell lines expressing HPV16 E6 isoforms

**Figure 2:**
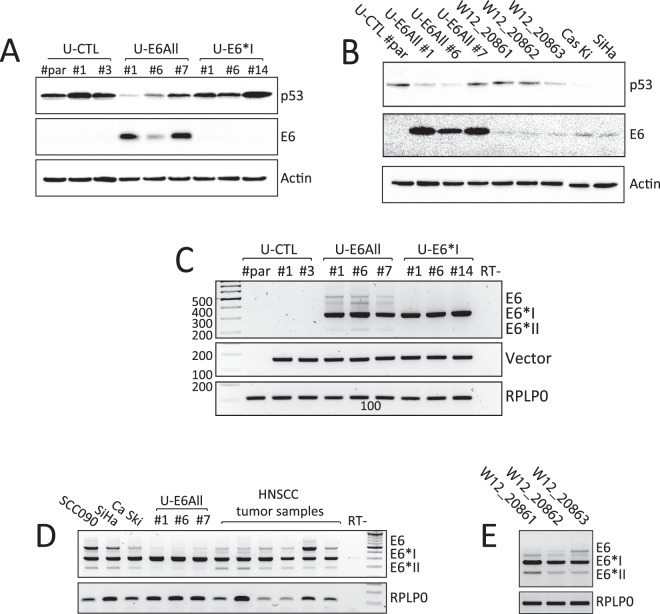
Expression profiles of HPV16 E6 and E6*I in stable cell lines. (**A**) Western blotting analysis showing p53, E6 and β-actin protein levels in U-2 OS parental cell line (U-CTL#par) and in stably transfected cells with pXJ40 empty vector (U-CTL #1 and #3) or pXJ40-E6All vector (U-E6All #1, #6 and #7) or pXJ40-E6*I vector (U-E6*I #1, #6 and #14). β-actin is used as loading control. Full-length blots are presented in Figure S4. (**B**) Western blotting analysis comparing protein levels of E6 and p53 between U-2 OS cellular clones (U-CTL#par, U-E6All #1, #6 and #7) and naturally HPV16-infected cells (W12_20861, W12_20862, W12_20863, SiHa and Ca Ski cell lines). β-actin is used as loading control. **(C)** RT-PCR analysis of alternatively spliced HPV16 E6 transcripts in U-E6All and U-E6*I stably transfected cells. As expected no E6 transcripts were observed in U-2 OS parental cell line (U-CTL#par) and in U-CTL #1 and #3 stably transfected cells. The detection of the vector in stably transfected cells was done by pXJ40 vector specific primers and RPLP0 was used as loading control. Full-length gels are presented in Figure S5. **(D)** RT-PCR analysis of alternatively spliced E6 transcripts in HPV16 + cell lines SCC090, SiHa, Ca Ski, in stable cell lines U-E6All #1, #6 and #7 and in HPV16 + HNSCC tumor samples. RPLP0 was used as loading control. Note that a band can be observed at 400 bp and could correspond to heteroduplexes between the different amplicons generated during PCR reaction. Full-length gels are presented in Figure S6. **(E)** RT-PCR showing alternatively spliced E6 transcripts in naturally HPV16-infected cells (W12_20861, W12_20862, W12_20863).

Stable cell lines expressing either all E6 isoforms or E6*I were generated from the U-2 OS cell line. Cellular clones were isolated upon selection of cells resistant to G418: U-CTL (clones #1 and #3, transfected with pXJ40-empty), U-E6All (clones #1, #6 and #7, transfected with pXJ40-E6All), and U-E6*I (clones #1, #6 and #14, transfected with pXJ40-E6*I). Then, these cellular clones were tested for E6 and E6*I expression. As expected, E6 protein was observed in U-E6All stable cell lines compared to U-CTL and U-E6*I. A decrease of the wild-type p53 protein level is observed in these cells, suggesting that U-E6All cells express of a functional E6 protein (Fig. 2A and S4A). Next, we compared E6 expression from U-E6All clones to naturally-infected HPV16 cell lines such as SiHa, Ca Ski and W12 clones. We observe a significantly higher expression of E6 in U-E6All clones. Interestingly, this high expression does not result in a greater degradation of p53 compared to SiHa and Ca Ski cells. We observe low amount of E6 in the three W12 clones tested but still managing variable levels of p53 between the three clones tested (Figs 2B and S4B). Because E6*I protein was hardly detectable, E6 and E6*I RNA levels were simultaneously monitored by RT-PCR. The RNA profile of U-E6All stable cell lines shows a low amount of full-length E6 RNA (unspliced) and E6*II RNA (SD226/SA556 spliced RNA) in comparison with the high amount of E6*I RNA (SD226/SA409 spliced RNA). As expected, U-E6*I stable cell lines express only E6*I RNA and U-CTL stable cell lines, like parental U-2 OS (U-CTL#par), did not express E6 or E6*I (Figs 2C and S5A). Similar splicing profiles of E6 transcripts are detected between U-E6All stable cell lines, HPV16-positive cancer cell lines (SCC90, SiHa, Ca Ski) and in most head and neck squamous cell carcinoma (HNSCC) tumor samples tested (Figs 2D and S5B). Also, in the naturally HPV16-infected cervical keratinocyte cells (W12 cells), E6*I transcript is the most abundant splice product compared to E6 and E6*II transcripts (Figs 2E and S5C).

### E6 and E6*I expression differentially alters phenotypic characteristics relevant to cancer cells

**Figure 3:**
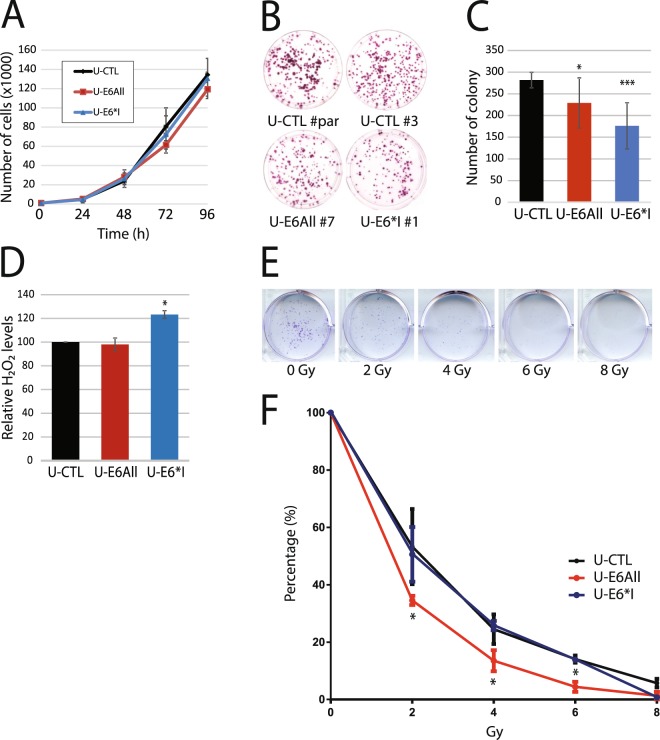
HPV16 E6 and E6*I expression differentially alters phenotypic characteristics relevant to cancer cells. (**A**) Cell proliferation assay with stably transfected cells U-CTL (black), U-E6All (red) and U-E6*I (blue) was performed to monitor daily live cells for 4 days. Cell counts are represented as mean +/− S.D. of 3 independent experiments. **(B)** Representative images of the colony formation assay realized with U-CTL#par, U-CTL#3, U-E6All#7 and U-E6*I#1. **(C)** Quantification of the colony formation assay realized with U-CTL (#par, #1, #3), U-E6All (#1, #6 and #7) and U-E6*I (#1, #6 and #14) cell lines. Foci counts are represented as mean +/− S.D. of 3 independent experiments. **(D)** Detection of H_2_O_2_ released from U-CTL (#par, #1, #3), U-E6All (#1, #6 and #7) and U-E6*I (#1, #6 and #14) cell lines in 1 h using the Amplex Red assay. H_2_O_2_ relative levels are represented as mean +/− S.D. of 3 independent experiments. **(E/F)** Clonogenic radio sensitivity assay performed with U-CTL, U-E6All and U-E6*I cell lines. Cell colonies (>50 cells) were counted 10 days following irradiation (0 to 8 Gy). Mann-Whitney test were used to assess statistical significance (*p < 0.05; **p < 0.01; ***p < 0.001).

To evaluate phenotypic characteristics relevant to cancer cells in our cellular models, functional assays were performed with U-CTL, U-E6All and U-E6*I cell lines. First, cell proliferation was assessed by measuring the number of cells once a day over 4 days. Results show that all cell lines had the same growth rate (Fig. 3A). In contrast, colony formation assays, performed to monitor the ability of a single cell to grow into a colony, show a significant decrease in the number of colonies for U-E6All compared with U-CTL. We observe that expression of E6*I alone has a stronger inhibitory effect on colony formation (Fig. 3B,C). E6 and E6*I from HPV16 have both been shown to induce oxidative stress in different cellular models^24,27^. Relative production and release of H_2_O_2_ in medium from cells, reported by the oxidation of amplex red reagent to fluorescent resorufin, was used as indicator of ROS production. We observe a 20% significant increase in H_2_O_2_ released from U-E6*I clones relative to control. However, no change in H_2_O_2_ release from U-E6All clones is observed (Fig. 3D). It was previously observed that HPV16 E6 increases the radio sensitivity of HPV-negative cancer cells^28^. To confirm this in our cellular models, the radiation sensitivity was measured *via* clonogenicity assays following irradiation. In all cell lines, survival decreases with increasing irradiation dose (Fig. 3E). We observed that only U-E6All cell lines show a significant decrease of survival after irradiation compared to U-CTL and U-E6*I cell lines (Fig. 3F). These data suggest that E6 and E6*I can have isoform-specific functions in the cells.

### Transcriptome profiles reveal that E6*I deregulates cellular targets independently of E6

**Figure 4:**
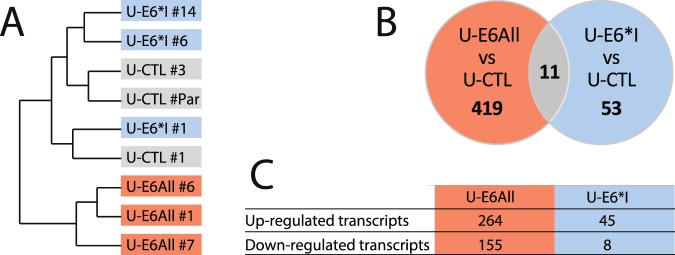
Transcriptome profiles reveal that E6*I deregulates cellular targets independently of E6. (**A)** Unsupervised hierarchical clustering of RNA-seq data from the U-CTL, U-E6All and U-E6*I cell lines. **(B)** Venn diagram showing the number of deregulated genes in the 2 pairwise comparisons U-E6All vs. U-CTL and U-E6*I vs. U-CTL. **(C)** Number of deregulated transcripts in U-E6All and U-E6*I cell lines compared to U-CTL.

To assess cellular targets potentially deregulated by E6*I, the whole transcriptome of stable cell lines – U-E6All (clones #1, #6 and #7), U-E6*I (clones #1, #6 and #14) and U-CTL (clones #1, #3 and #par) – were analyzed by RNA sequencing. First, unsupervised hierarchical clustering analysis based on RNA sequencing datasets reveals 2 clusters. One of these clusters contains the 3 clones of U-E6All cells (Fig. 4A and S6). Venn diagram representation of deregulated genes in U-E6All and U-E6*I compared with U-CTL show that 419 transcripts were significantly deregulated (FDR < 0.05) in U-E6All (Fig. 4B), of which 264 were upregulated and 155 were down-regulated (Fig. 4C and Table S1). The expression of 53 transcripts were deregulated in U-E6*I cell lines (Fig. 4B), of which 45 were up-regulated and 8 were down-regulated (Fig. 4C and Table S2). An overlap of 11 deregulated transcripts was observed between U-E6All and U-E6*I cell lines. These data suggest that E6*I modulates cellular gene expression independently of E6 but also indicate that the effect of E6*I on transcriptome is disrupted when co-expressed with E6.

### GO terms and KEGG pathway analysis of deregulated transcripts in U-E6All and U-E6*I cells

**Figure 5:**
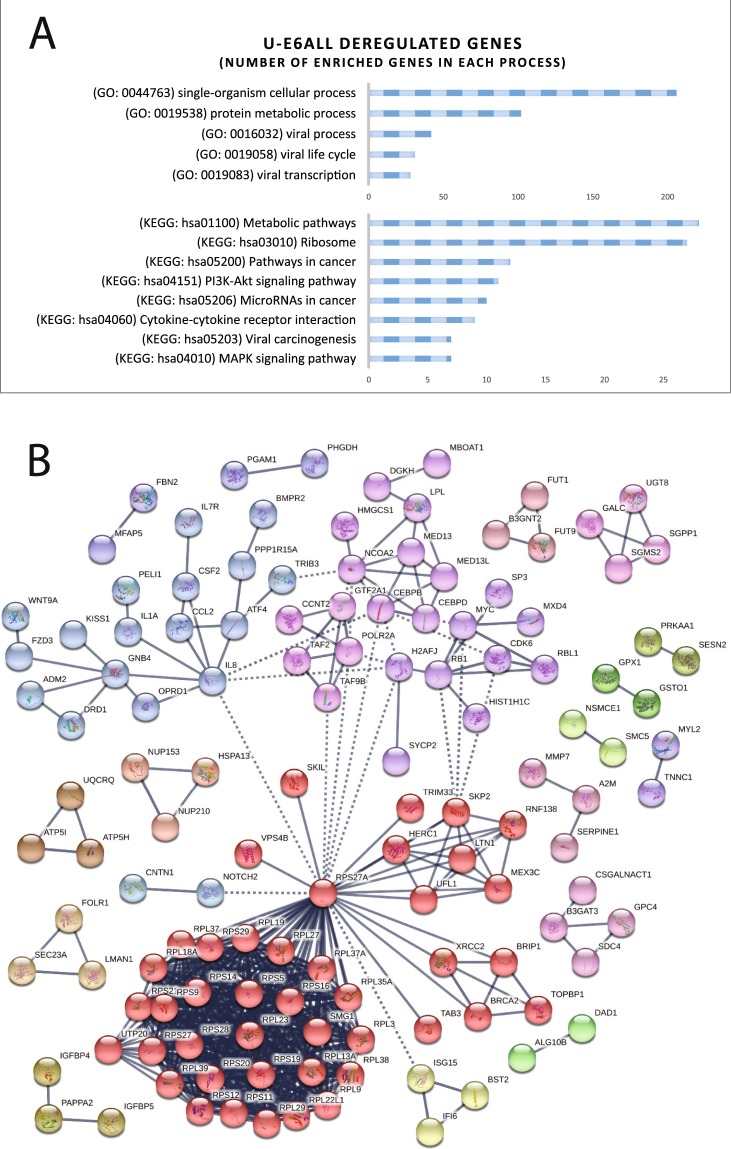
Enrichment and pathway analysis of deregulated genes in U-E6All cell lines compared with U-CTL. (**A)** Gene Ontology and KEGG pathway analysis of differentially expressed transcripts in U-E6All cell lines. **(B)** Protein-protein interaction network, corresponding to the protein-encoding genes deregulated in U-E6All cell lines, generated using STRING database^75^. Line thickness indicates the strength of data support.

Classification using GO (Gene Ontology) enrichment and KEGG (Kyoto Encyclopedia of Genes and Genomes) pathway analysis showed an over-representation of U-E6All deregulated transcripts in enriched terms, such as single-organism cellular process (GO: 0044763), protein-metabolic process (GO: 0019538), metabolic pathways (KEGG: hsa01100), ribosome (KEGG: hsa03010) and pathways in cancer (KEGG: hsa05200) (Fig. 5A). Based on protein-coding transcripts deregulated in U-E6All cells, a protein interaction network was constructed using the STRING database (Fig. 5B). Many of the deregulated transcripts identified in U-E6All cells have been previously identified in HPV-infected cells and tumor samples such as transcripts encoding ribosomal subunits, SYCP2, NUP210 and DICER1^29–33^.

**Figure 6:**
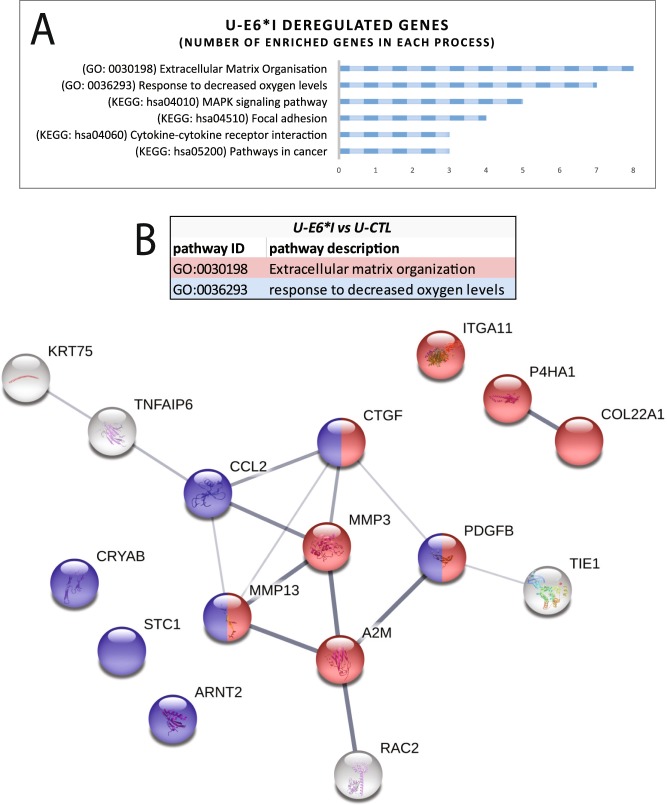
Enrichment and pathway analysis of deregulated genes in U-E6*I cell lines compared with U-CTL. (**A)** Gene Ontology and KEGG pathway analysis of differentially expressed transcripts in U-E6*I cell lines. **(B)** Protein-protein interaction network, corresponding to the protein-encoding genes deregulated in U-E6*I cell lines, generated using STRING database^75^. Line thickness indicates the strength of data support. In blue and red are represented proteins involved in extracellular matrix organization and response to decreased oxygen levels, respectively.

Gene ontology and KEGG pathway analysis of U-E6*I cell lines revealed 6 gene classes deregulated by ectopic expression of E6*I: Extracellular Matrix Organization (GO:0030198), Response to decreased oxygen levels (GO: 0036293), MAPK signaling pathway (KEGG: hsa04010), Focal adhesion (KEGG: hsa04510), Cytokine-cytokine receptor interaction (KEGG: hsa04060) and Pathway in cancer (KEGG: hsa05200) (Fig. 6A). In addition, the STRING database reported interactions between 10 protein-coding transcripts based on text mining, protein-protein interaction and co-expression data. Furthermore, these data show that the core interactors belong to the extracellular matrix organization and/or oxidative stress response pathways (Fig. 6B). These data reveal specific pathways deregulated by E6*I in an E6-independent manner.

### RNA sequencing transcripts validation by RT-qPCR

**Figure 7:**
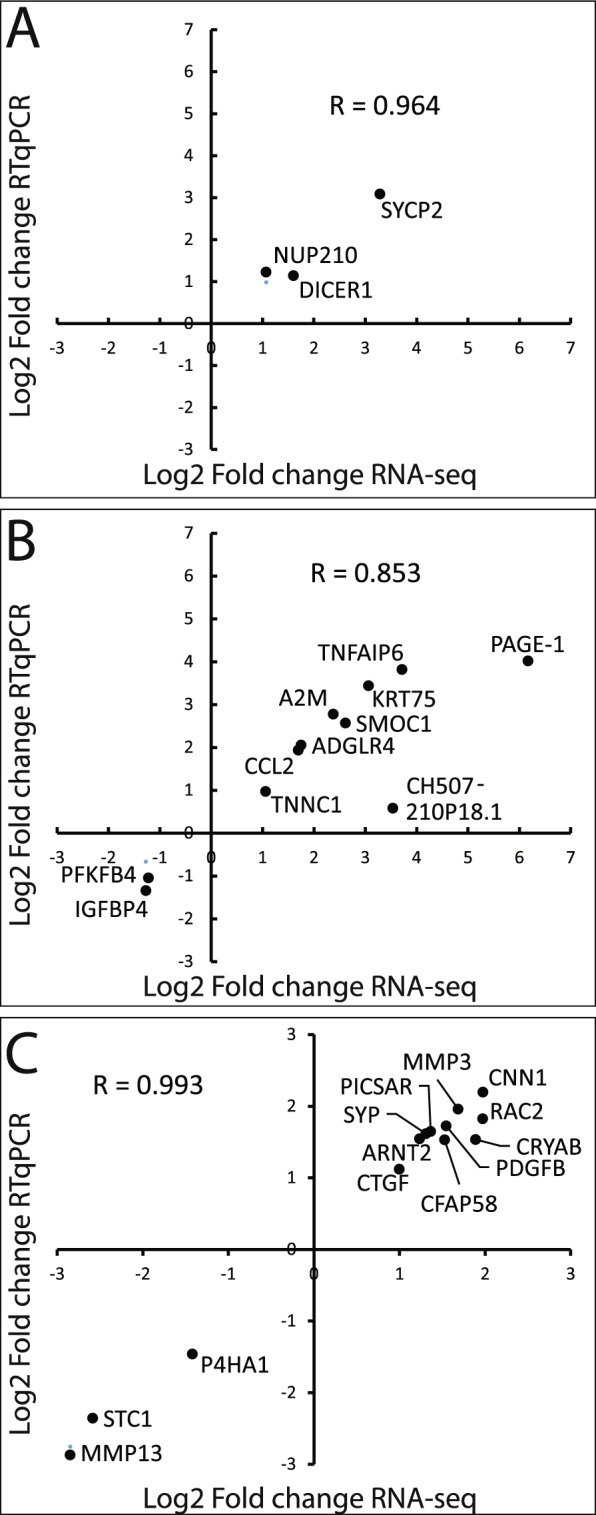
Correlation between RNA-sequencing and RT-qPCR results. RT-qPCR analyses of **(A)** 3 transcripts deregulated in U-E6All cell lines (U-CTL vs U-E6All), **(B)** the 11 transcripts deregulated in both U-E6All and U-E6*I cell lines (U-CTL vs U-E6*I) and **(C)** 13 transcripts deregulated in U-E6*I cell lines (U-CTL vs U-E6*I). Fold changes are represented in x- and y-axes for RNA-seq and RT-qPCR, respectively. For each condition, the data represent mean of the 3 clones. For RT-qPCR, RPLP0 was used as housekeeping gene.

To validate RNA-seq data, expression of several transcripts was confirmed by RT-qPCR. We selected SYCP2, NUP210 and DICER1 transcripts deregulated in U-E6All cells and already known to be modulated by E6 expression (Fig. 7A), the 11 transcripts deregulated in both U-E6All and U-E6*I cells (Fig. 7B) and 13 transcripts only deregulated in U-E6*I cells including the 6 transcripts assigned to “response to decreased oxygen levels” (GO: 0036293) (Fig. 7C). Except for the lincRNA (long intergenic non-coding RNA) named CH507-210p18.1, fold changes estimated by RT-qPCR and RNA-sequencing are consistent for all transcripts tested. Pearson correlation coefficients (R) were calculated. We found high correlations (R = 0.996 for U-E6All-deregulated transcripts, R = 0.853 for both U-E6All- and U-E6*I-deregulated transcripts and R = 0.993 for U-E6*I-deregulated transcripts) indicating a strong concordance between RNA-seq and RT-qPCR data.

### Expression pattern of E6 or E6*I deregulated transcripts in W12 cell line

**Figure 8:**
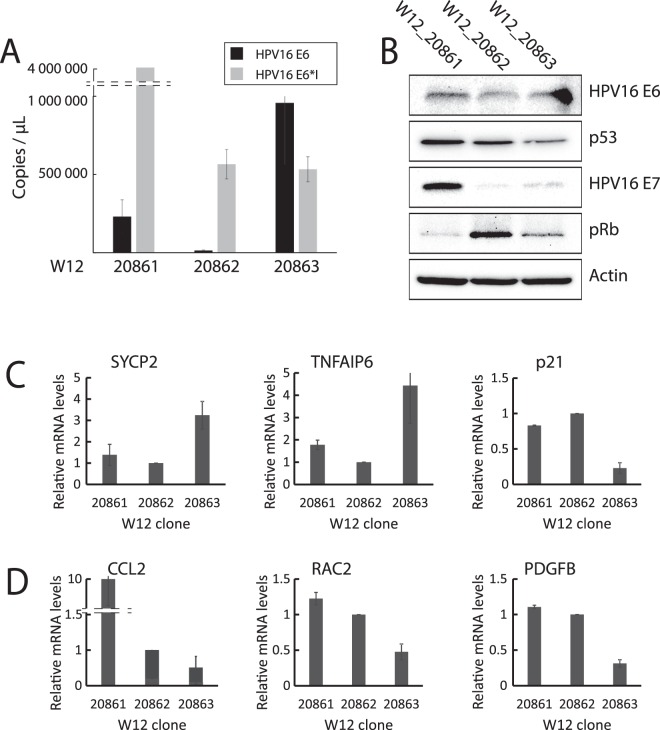
Expression levels of E6- and E6*I-deregulated transcripts in W12 clones. (**A)** RT-qPCR analysis showing E6 and E6*I absolute mRNA levels (copies per µL of pure cDNA) in W12_20861, W12_20862 and W12_20863 clones. pXJ40-E6 and pXJ40-E6*I diluted in salmon sperm DNA (50 ng/µL) were used to generate standard curves. **(B)** Western blot analysis showing HPV16 E6 and E7, p53 and pRb protein levels in W12 clones. β-actin is used as loading control. RT-qPCR analyses showing. Full-length gels are presented in Figure S7. **(C)** SYCP2, TNFAIP6 and p21, and **(D)** CCL2, RAC2 and PDGFB relative mRNA expression in W12 clones. RPLP0 was used as housekeeping gene. RT-qPCR are represented as means +/− S.D. of 3 independent experiments.

To validate the biological significance of the cellular transcripts found deregulated by E6*I, we performed experiments on W12 cells, a naturally HPV16-infected cervical keratinocyte cell line. First, absolute quantification was done to measure the copy number of E6 and E6*I mRNAs in three undifferentiated W12 clones (W12_20861, W12_20862, W12_20863). The W12_20861 clone expresses the highest level of E6*I transcript (approximately 4.10^6^ copies/µL) compared to W12_20862 and W12_20863 clones (approximately 500 000 copies/µL) (Fig. 8A). Unspliced E6 transcripts are highly expressed in the W12_20863 clone (approximately 1.10^6^ copies/µL) compared to W12_20861 and W12_20862 clones (approximately 230 000 and 14 000 copies/µL, respectively). In each W12 clone tested, we observed a strong correlation between RNA and protein levels for E6 with high level of E6 protein and low level of p53 protein in W12_20863 clone compared to W12_20861 and W12_20862. The high levels of E6*I mRNA and E7 protein in W12_20861 clone is also consistent with the lowest level of pRB protein (Figs 8B and S7). Overall, each clone possesses a unique expression profile of HPV transcripts and proteins allowing us to correlate those profiles to the expression of cellular targets identify by RNA-seq. So, the expression levels of transcripts modulated by E6 such as SYCP2, p21 and TNFAIP6 was measured by RT-qPCR in each W12 clone. As expected, SYCP2 and TNFAIP6 transcripts are upregulated while p21, a p53 target gene, is downregulated in the W12_20863 clone expressing the highest level of E6 (Fig. 8C).

Next, we monitored the RNA levels of CCL2, RAC2 and PDGFB transcripts modulated by E6*I. These transcripts encode proteins implicated in the activation of the NADPH Oxidase complex-2 (Nox2), required for Nox-dependent ROS production. There were higher levels of CCL2 and RAC2 in W12 clone expressing high level of E6*I (W12_20861). Nevertheless, a decreased expression of these transcripts is observed in cells expressing high level of E6 (W12_20863) suggesting that E6 could have the ability to counteract the effects of E6*I. Altogether, these results show that E6*I expression could drive the expression of genes implicated in oxidative stress metabolism in the W12 cell lines but also indicate that E6 can abrogate the effect of E6*I on ROS metabolism.

## Discussion

To our knowledge, this study is the first report assessing the impact of HPV16 E6*I on cellular gene expression using RNA sequencing. The aim of this study was to uncover target genes specifically modulated by E6*I independently of the full length E6 protein. To this end, stable cell lines expressing either all E6 protein isoforms (U-E6All) or E6*I (U-E6*I) exclusively, were generated from U-2 OS. The U-2 OS cell line represents an adequate model for studying HPV gene expression and HPV-associated carcinogenesis because it expresses a wild-type p53, which can be targeted by E6. Furthermore, it was shown that viral gene expression of both alpha and beta papillomaviruses in U-2 OS is very similar to that described in keratinocytes^34^. Immunoblotting experiments allowed to confirm the expression of a functional E6 by loss of p53 in U-E6All cell lines^35^. E6 early transcripts were also efficiently spliced in U-E6All cell lines, yielding high amount of E6*I transcripts as observed in HPV16-positive cell lines and HNSCC clinical samples^36^. However, although the E6*I protein was detected in transient transfection, no protein was observed in stable cell lines, probably due to the extremely short half-life of the E6*I isoform. This was already documented in a previous study, which reported the detection of an HPV16 E6*I fusion protein following transient transfection but failed to detect E6*I in stable cell lines^37^. In the present study, we observe an accumulation of endogenous p53 protein in the HPV16 E6*I-expressing U-2 OS cells, suggesting that E6*I could have opposite effect of E6 on p53 protein level. But, we also show that E6*I expression causes an accumulation of ROS, indicating that the increasing level of p53 observed can be mediated by the ROS production. Also, emerging roles of non-coding RNAs in gene expression regulation and the noticeable difficulties to detect the E6*I protein by western blotting encouraged us to consider that the transcriptome deregulation observed in the present study could be mostly mediated by HPV16 E6*I RNA rather than the E6*I protein^38^. This information will raise exciting and challenging questions for understanding HPV-mediated carcinogenesis.

To assess the biological characteristics of the stable cell lines, we carried out several functional assays. We confirm that E6 isoforms or E6*I expression in HPV-negative cells had no impact on cell proliferation^20,39^. A previous study^40^ has observed that E6 expression inhibited colony formation. Here, we show that E6*I had a stronger inhibitory effect on colony formation than E6. Congruent with a previous study using U-2 OS cells transiently expressing E6 isoforms or only E6*I^24^, we report that stable expression of E6*I increases ROS production but no change is observed in cells co-expressing E6. Finally, radio sensitivity assays showed that U-E6All cells are more sensitive to radiation than both E6*I and control cells. These latter results could be partially explained by the *TP53* wild-type status of our cellular model given the fact that E6 was shown to disrupt the p53-mediated cellular responses to DNA damage^41^. Altogether, these data suggest that E6 isoforms have a specific impact on cellular phenotype and that the impact of E6*I is modulated when co-expressed with E6.

Recently, a whole transcriptome study using differentiated keratinocytes showed that HPV16 infection modulates genes involved in the regulation of cellular matrix adhesion, ROS biosynthesis and inflammatory response^42^. In our study, RNA sequencing identified 419 transcripts significantly deregulated in HPV-negative cellular model expressing E6 isoforms. Among them, a substantial number was already described in the literature as deregulated in HPV16 cellular models or in HPV16-positive clinical samples such as the down-regulation of a large set of transcripts encoding ribosomal subunits^29^. Hence, KEGG pathway and GO enrichment analysis showed viral gene expression and protein synthesis related terms enriched. Our HPV-negative U-2 OS cellular model highlights the implication of E6 in the downregulation of ribosomal subunits transcripts. In addition, we confirm the upregulation of SYCP2, Nup210 and DICER1 transcripts, which were previously shown to be increased in HPV16-positive clinical samples from cervical^29^ and head and neck cancers, and HPV-negative cell lines expressing E6 and E7 proteins^30,33^.

Comparative RNA sequencing analysis also revealed that E6*I expression was sufficient to modulate the cellular transcriptome. KEGG pathway and GO enrichment annotations showed that E6*I-deregulated transcripts were assigned to extracellular matrix organization, response to decreased oxygen levels, mitogen activated protein kinase (MAPK), focal adhesion, cytokine-cytokine receptor interaction and cancer pathways. STRING analysis indicates that most transcripts implicated in extracellular matrix and response to decreased oxygen levels are predicted to form a significantly enriched core of interactors. A previous study, as well as ROS measurement in our model, indicate that E6*I increases levels of ROS^24^. Thus, we focused downstream analyses on E6*I-deregulated transcripts implicated in the latter ontology, namely CRYAB^43^, CCL2^44^, CTGF^45^, PDGFB^46,47^, STC1^48^, ARNT2^49^, MMP13^50,51^. MMP3^52,53^, RAC2^54,55^ and TNFAIP6^56,57^ were also found associated with ROS metabolism. These E6*I-deregulated genes have been validated using RT-qPCR. The high correlations found demonstrate the robustness of fold changes estimated from RNA-seq.

To further investigate the effects of E6*I expression observed in the U-2 OS model, we validated the cellular target genes involved in ROS metabolism in the W12 HPV16-infected keratinocyte cell line. These cells, derived from a low-grade cervical lesion, has been extensively used for the study of HPV infection, life cycle and carcinogenesis. Many clones have been established harboring episomal or integrated type I and II HPV16 genomes to study all those aspects^58^. Interestingly, each clone expresses different levels of E6 and E6*I mRNAs allowing us to get a better understanding on how E6*I potentially modulates the cellular targets identified by RNA-seq and on the existing interplay between E6 and E6*I. Quantification of HPV16 transcripts and proteins in W12 clones revealed a close correlation between mRNA levels and subsequent protein levels as it has been previously reported^59^. TNFAIP6 was found upregulated in both U-E6All and U-E6*I cells by RNA-seq. Nevertheless, TNFAIP6 RNA levels correlate with E6 expression in the W12. Finally, we confirm that RAC2, CCL2 and PDGFB mRNA levels correlate with E6*I expression in the W12 cellular model. RAC2 is a Rho-like small GTPase, implicated in the activation of the NADPH Oxidase complex-2 (Nox2), catalyzing the production of ROS. It has been shown that ROS production induced by Rac2 leads to DNA damage in leukemia stem cells causing genome instability suspected to drive the rapid accumulation of mutations in progenitor cells^54^. Low levels of RAC2 have also been linked to lower ROS production and DNA damage in quiescent cells exposed to radiation^55^. Interestingly, PDGF growth factor is also closely related to the Nox complex. PDGF interaction with its receptor was shown to activate ROS production by the Nox complex in hepatic^47^ and epithelial model^46^. Finally, CCL2 increases production of ROS by regulating gp91phox, a subunit of the Nox complex^60^. CCL2 expression has also been shown to be reduced when the Nox complex is impaired and produce low levels of ROS^61^. The expression of RAC2, CCL2 and PDGFB induced by E6*I could drive Nox2 complex activation, thus representing a new pathway by which E6*I is able to increase cellular ROS production.

Oxidative stress is crucial in carcinogenesis because of its role in inflammation, mutagenesis, initiation and neoplastic progression^62^. Cellular antioxidant defenses exist to maintain cellular homeostasis and viruses are well known to induce oxidative stress by interfering with those defenses^63,64^. Studies have linked HPV16 E6*I expression to changes in the activity of antioxidant enzymes, increased level of ROS and accumulation of DNA damage^24,65,66^. In our study, RNA sequencing did not reveal any change in SOD and GPX mRNA levels when E6*I is expressed, probably due to a post-translational regulation of these enzymes by E6*I. In the context of hrHPV-driven carcinogenesis, it has been proposed that oxidative stress induced by E6*I could cause genome instability thus facilitating the integration of HPV genomes into the host cell genome^67,68^. This hypothesis is consistent with the correlation between the severity of cervical lesions and the increasing levels of spliced E6*I mRNA^5,12–14,26^.

In conclusion, our study reports that HPV16 E6*I induces modulation of the U-2 OS cellular transcriptome using RNA sequencing. We identified cellular genes involved in ROS metabolism that were deregulated by the ectopic expression of E6*I in our model and that are also found deregulated by endogenous E6*I expression in the W12 keratinocyte model. In agreement with previous studies, E6*I can promote viral genome integration into host genome^69^ by inducing ROS and subsequently increasing genome instability^24^. Here, we highlight the NADPH oxidase pathway as a new potential target of HPV16 E6*I that could explain the increased ROS production. Further studies will be needed to fully understand how oxidative stress related genes modulated by E6*I could affect HPV-driven carcinogenesis and life cycle. This study also demonstrate that E6*I-cellular transcriptome modulation is altered in the presence of E6 suggesting, among others things, a rivalry between the two protein isoforms. The overlap of only 11 transcripts between U-E6All and U-E6*I conditions suggests that E6 is able to partially negate the effects of E6*I. The modulation of ROS production in U-2 OS, and ROS-related genes expression in U-2 OS and W12 clones depending on E6/E6*I levels is in agreement with this interplay. Similarly, it has been observed that the effects of ectopic E6*I on antioxidant enzymes^24^ seems to be lost when E6*I is co-expressed with E6^70^. Other paradoxical observations were made concerning the expression of E6 and E6*I. The first aspect of this interplay was reported on the opposite effects of both isoforms on p53 degradation and subsequent apoptosis of E6-expressing cells^20^. More recently, the protection of E6-expressing cells from TNF was only seen when E6 or E6*I are expressed alone but not when both isoforms are expressed^37^. These observations ask the question of how E6*I can be considered separately of E6 in the natural history of HPV16 infection.

## Materials and Methods

### Cell culture

Human osteosarcoma derived cell line U-2 OS, HPV16-positive carcinoma derived cell lines SiHa (ATCC® HTB-35™), SCC090 (ATCC® CRL-3239™) and U-2 OS stable cell lines were cultured in DMEM medium (Lonza, Verviers, Belgium). U-2 OS stably transfected clones were routinely cultured in the presence of 625 μg/mL G418 to maintain selection. HPV16-positive carcinoma derived cell line Ca Ski (ATCC® CRL-1550™) was cultured in RPMI-1640 medium (Lonza, Verviers, Belgium). All media were supplemented with 10% Fetal Calf Serum (Eurobio, Courtaboeuf, France). Undifferentiated W12 20861, 20862 and 20863 clones were cultured in monolayer on Mitomycin C treated mouse 3T3 cells as previously described^58^. Cell lines were incubated at 37 °C in a humidified atmosphere with 5% of CO_2_.

### Plasmids, transient and stable transfections

pXJ40 (empty) and pXJ40-E6All vectors, containing the HPV16 E6 coding sequence, have been generously provided by Murielle Masson (ESBS, Illkirch, France). The pXJ40βGlo∆int vector was generated from the pXJ40 vector, using Q5Site-Directed Mutagenesis Kit (New England Biolabs) and the following primers 5′-CTCCTGGGCAACGTG-3′ and 5′-CCTGAAGTTCTCAGGATCG-3′. The pXJ40-E6*I vector, lacking the intron 1 located in the E6 ORF corresponding to the nucleotides from 227 to 408 was generated from the pXJ40-E6All vector (Primers: 5′-GTGTATTAACTGTCAAAAGCC-3′ and 5′-CTCACGTCGCAGTAACTG-3′). Transient transfections of the U-2 OS cell line were performed using JetPEI reagent (Polyplus-transfection, Illkirch, France) according to manufacturer’s instructions. Cells were incubated in the presence or absence of 10 µM of proteasome inhibitor MG132 for 16 h and harvested after 48 h incubation post-transfection. U-2 OS stable cell lines were obtained using JetPEI reagent by co-transfecting with a ratio of 1:9 respectively, the pcDNA3.1-2Flag vector (neomycin resistance) and corresponding pXJ40 vectors (pXJ-CTL, pXJ40-E6All and pXJ40-E6*I). G418 resistant stable clones were then selected after 3 weeks under selection with 800 µg/mL G418 (Euromedex).

### Cell proliferation and clonogenicity assays

Cell proliferation assays were done with 10,000 cells in 12-well plates by counting cells each day for 4 days using the EVE^TM^ automatic cell counter (Nano EnTek). Clonogenicity assays were performed by seeding 500 cells of each U-CTL, U-E6All and U-E6*I cell lines in 10 cm dishes. After 14 days of culture, colony were counted after crystal violet staining. Clonogenicity radio sensitivity assays were performed by seeding 300 cells in 6-well plates 24 h before gamma irradiation (0 to 8 Gy) using Gammacell® 40 Exactor (Best Theratronics, Ltd). After treatment, cells were cultured for 10 days then stained with crystal violet. Cell colonies presenting >50 cells were finally counted. For each biological replicate (n = 3), 2 wells were analyzed for colonies counting.

### Measurement of Hydrogen peroxide (H_2_O_2_)

Measurement of H_2_O_2_ released from stably transfected U-2 OS cells was carried using the Amplex® Red Hydrogen Peroxide/Peroxidase Assay Kit (Invitrogen) following manufacturer instructions. Four hours after seeding 800,000 cells of each clone in 35 mm dishes, cells were incubated for 1 h in 500 µL PBS1X. Then, 50 µL was transferred in an opaque 96 well plate in triplicate and mixed with an equal amount of Amplex Red reagent (50 µM Amplex Red and 0,1 U/mL HRP final concentrations). After 1 h incubation at room temperature in the dark, fluorescence was measured (560_ex_/590_em_ nm) on a Synergy^TM^ H1 microplate reader (Biotek).

### Immunoblot assays

U-2 OS, SiHa, Ca Ski or W12 cells were lysed using RIPA buffer (50 mM Tris-HCl pH7.4, 150 mM NaCl, 1% NP40, 0.5% Na deoxycholate, 1 mM EDTA) supplemented with 30 µg/mL of anti-protease mixture (Roche Diagnostics) followed by sonication. After centrifugation at 10,000 × *g* for 10 min at 4 °C, protein concentrations were determined in supernatant using Bio-Rad Protein Assay (Bio-Rad) according to manufacturer’s instructions. Forty µg of protein in Laemmli lysis buffer were then loaded onto a sodium dodecyl sulfate-polyacrylamide gel electrophoresis (SDS-PAGE) gel. After the transfer of proteins onto 0.2 µm Amersham^TM^Hybond^TM^-PVDF membrane (GE Healthcare) and blocking 1 hour with 5% nonfat milk in Tris-buffered Saline-Tween20, membranes were probed with primary antibodies for 2 hours (except cell signaling antibodies on night) then with HRP-conjugated secondary antibodies (see below) for 1 h. Chemiluminescent signal was detected using PierceECL 2 Western Blotting Substrate (ThermoScientific).

### Antibodies

Anti-HPV16E6 antibodies 1E-6F4 (E6 N-ter) and 2E-3F8 (E6 C-ter) were obtained from Euromedex. Anti-p53 (DO-1) and anti-pRb (4H1) antibodies were obtained from Cell Signaling. Anti-β-actin (AC-15) antibody was obtained from Sigma-Aldrich. Anti-HPV16E7 (NM2) antibody was obtained from Santa Cruz Biotechnology. HRP-conjugated goat anti-mouse secondary antibody was purchased from BD Pharmingen. Primary and secondary antibodies have been diluted respectively to 1:1000 and 1:5000 in blocking buffer (except for β-actin at 1:20,000 and 1:30,000).

### RT-PCR and RT-qPCR

Total RNAs were extracted using Ribozol reagent (Amresco) and then 500 µg were retro-transcribed into cDNA using Maxima first strand cDNA synthesis kit (ThermoScientific) according to manufacturer instructions. For PCR, cDNAs were amplified using DreamTaq polymerase (Thermo Scientific) according to manufacturer’s instructions. PCR products were analyzed on a 2% (w/v) agarose gel prepared with Tris-Borate-EDTA buffer. Quantitative PCR was performed using Power SYBR Green PCR mix (Life Technologies) according to manufacturer instructions and analyzed on a StepOnePlus Real-Time PCR system (Applied Biosystems). Serial dilutions of pXJ40-E6 and pXJ40-E6*I from 10^7^ to 10^1^ copies/µL diluted in salmon sperm DNA (50 ng/µL) (Invitrogen) were used as template to generate standard curve for absolute quantification of E6 and E6*I mRNA copies respectively in W12 clones. Primer sequences used are listed in Supplementary Table 3. RPLP0 expression levels was used as internal normalization standards. The 2^−ΔΔCt^ method was used for relative mRNA quantification.

### RNA sequencing

RNA-seq was carried out to identify differentially expressed transcripts in U-E6All or U-E6*I cell lines compared to U-CTL cell lines. For each clone, total RNAs were extracted using Ribozol reagent (Amresco) according to manufacturer’s instructions. cDNA libraries were produced with 1 µg of total RNA using TrueSeq Stranded mRNA Sample preparation kit (Illumina, Inc.). Sequencing was carried on 75 bp in single read on the NextSeq500 platform using the NextSeq500 High Output v2 kit (Illumina, Inc.).

### Reads alignment

Raw reads quality was assessed using the fastQC software v0.11.3 (Babraham Institute, UK). Reads from each sample were then aligned to the reference human genome (GRCh38, release 87) using TopHat v2.1.1 (John Hopkins University, USA)^71^ with a minimum mapping quality score of 30. For each sample, an average of 45 million reads were uniquely mapped.

### Differential expression analysis of cellular transcripts deregulated by E6 and/or E6*I

Cufflinks (Trapnell Laboratory, University of Washington, USA) was used for transcripts assembly and to assess transcripts abundance, which was normalized between all 9 samples using the fragments per kilobase per million mapped reads (FPKM) method to correct transcript length and library size bias^72^. Differentially expressed transcripts between control condition U-CTL and U-E6All or U-E6*I conditions were finally identified using Cuffdiff (Trapnell Laboratory). Transcripts with a false discovery rate (FDR) < 0,05 were considered significantly deregulated. No fold change cutoff was applied to the data. Nevertheless, more than 95% of transcripts with FDR < 0,05 had a fold change >2.

### Accession numbers

RNA sequencing data in the form of BAM files have been submitted to SRA@ncbi.nih.gov under the BioProject PRJNA421890 and BioSamples SAMN08159315 (U-CTL #par); SAMN08159316 (U-CTL #1); SAMN08159317 (U-CTL #3); SAMN08159318 (U-E6All #1); SAMN08159319 (U-E6All #6); SAMN08159320 (U-E6All #7); SAMN08159321(U-E6*I #1); SAMN08159323 (U-E6*I #6); SAMN08159324 (U-E6*I #14).

### Functional analysis of deregulated genes

Enrichment analyses were performed using Gene Ontology (GO) database v1.2 on PANTHER v13.0^73^, Kyoto Encyclopedia of Genes and Genomes (KEGG) release 81.0^74^ and STRING v10.5^75^.

### Statistics

Statistical analyses were carried out using XLSTAT v2017.4 (Addinsoft, Paris, France). Pearson’s correlation coefficients (r) were used to investigate correlation between RNA sequencing and RT-qPCR analyses. Mann-Whitney tests were used to analyze statistical significance of proliferation, clonogenicity and H_2_O_2_ assays, and RT-qPCR experiments (*p < 0.05; **p < 0.01; ***p < 0.001). Proliferation, clonogenicity, H_2_O_2_ and RT-qPCR graphs represent the mean of 3 experiments and the error bars represent the standard deviation.

## Supplementary information

Supplementary Information

Supplementary Table 1

Supplementary Table 1

Supplementary Table 3
