# Supplementary material for: Comparative RNA sequencing reveals that HPV16 E6 abrogates the effect of E6*I on ROS metabolism
Source: Sci Rep. 2019 Apr 11;9:5938. doi: 10.1038/s41598-019-42393-6 (PMC6459911; doi:10.1038/s41598-019-42393-6)
Supplement: Supplementary file 1 — Supplementary Information [file 41598_2019_42393_MOESM1_ESM.pdf]

Supplementary Information

Comparative RNA sequencing reveals that HPV16 E6 abrogates the effect of E6*I on ROS metabolism

Philippe Paget-Bailly[1,2], Koceila Meznad[1,2], Diane Bruyère[4], Jérôme Perrard[1,2], Michael Herfs[4], Alain Jung[5], Christiane Mougin[1,2], Jean-Luc Prétet[1,2,3], Aurélie Baguet[1,2*]

[1] EA3181, LabEx LipSTIC ANR-11-LABX-0021, UFR Santé, 19 rue Ambroise Paré, Besançon.

[2] Université Bourgogne Franche Comté, France.

[3] Centre Hospitalier Régional Universitaire, CNR HPV, 3 Bvd Alexandre Fleming, Besançon, France.

[4] Laboratory of Experimental Pathology, GIGA-Cancer, University of Liege, Liege, Belgium.

[5] Université de Strasbourg, Inserm, UMR_S1113, Centre de lutte contre le cancer Paul STRAUSS, Strasbourg, France.

[*]Corresponding author : Aurélie Baguet, UFR Santé, EA 3181 - Carcinogenèse associée aux HPV, 19 rue Ambroise Paré, 25000 Besançon, France. Tel : +33 3 63 08 22 65. E-mail : aurelie.baguet@univ-fcomte.fr

A

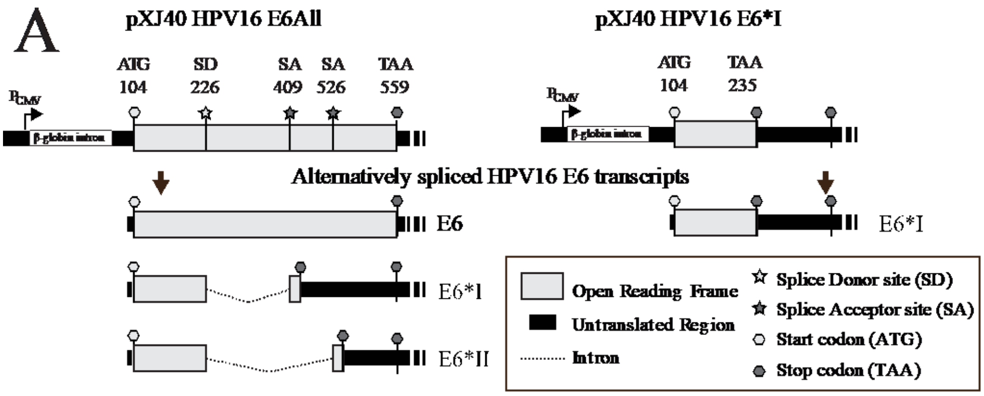

B

```
E6      104 ATGTTTCAGGACCCACAGGAGCGACCCAGAAAGTTACCACAGTTATGCACAGAGCTGCAA
          1  M  F  Q  D  P  Q  E  R  P  R  K  L  P  Q  L  C  T  E  L  Q

            ACAACTATACATGATATAATATTAGAATGTGTGTACTGCAAGCAACAGTTACTGCGACGT
             T  T  I  H  D  I  I  L  E  C  V  Y  C  K  Q  Q  L  L  R  R

            GAGGTATATGACTTTGCTTTTCGGGATTTATGCATAGTATATAGAGATGGGAATCCATAT
             E  V  Y  D  F  A  F  R  D  L  C  I  V  Y  R  D  G  N  P  Y

            GCTGTATGTGATAAATGTTTAAAGTTTTATTCTAAAATTAGTGAGTATAGACATTATTGT
             A  V  C  D  K  C  L  K  F  Y  S  K  I  S  E  Y  R  H  Y  C

            TATAGTTTGTATGGAACAACATTAGAACAGCAATACAACAAACCGTTGTGTGATTTGTTA
             Y  S  L  Y  G  T  T  L  E  Q  Q  Y  N  K  P  L  C  D  L  L

            ATTAGGTGTATTAACTGTCAAAAGCCACTGTGTCCTGAAGAAAAGCAAAGACATCTGGAC
             I  R  C  I  N  C  Q  K  P  L  C  P  E  E  K  Q  R  H  L  D

            AAAAAGCAAAGATTCCATAATATAAGGGGTCGGTGGACCGGTCGATGTATGTCTTGTTGC
             K  K  Q  R  F  H  N  I  R  G  R  W  T  G  R  C  M  S  C  C

            AGATCATCAAGAACACGTAGAGAAACCCAGCTGTAA 559
             R  S  S  R  T  R  R  E  T  Q  L  *  151
```

C

```
E6*I    104 ATGTTTCAGGACCCACAGGAGCGACCCAGAAAGTTACCACAGTTATGCACAGAGCTGCAA
          1  M  F  Q  D  P  Q  E  R  P  R  K  L  P  Q  L  C  T  E  L  Q

            ACAACTATACATGATATAATATTAGAATGTGTGTACTGCAAGCAACAGTTACTGCGACGT
             T  T  I  H  D  I  I  L  E  C  V  Y  C  K  Q  Q  L  L  R  R

            GAGGTGTATTAActgtcaaaagccactgtgtcctgaagaaaagcaaagacatctggacaa
             E  V  Y  *  43

            aaagcaaagattccataatataaggggtcggtggaccggtcgatgtatgtcttgttgcag

            atcatcaagaacacgtagagaaacccagctgtaa
```

Figure S1. HPV16 E6 cDNA sequences cloned into pXJ40 vector and their encoding capacity. (A) Schematic representation of HPV16 E6 and E6*I constructs used in our study. The expression of HPV16 E6 vector can generate by alternative splicing E6, E6*I and E6*II transcripts (left panel). The expression of HPV16 E6*I vector can only produce E6*I transcripts (right panel). (B) E6 coding sequence cloned into pXJ40. (C) E6*I coding sequence cloned into pXJ40.

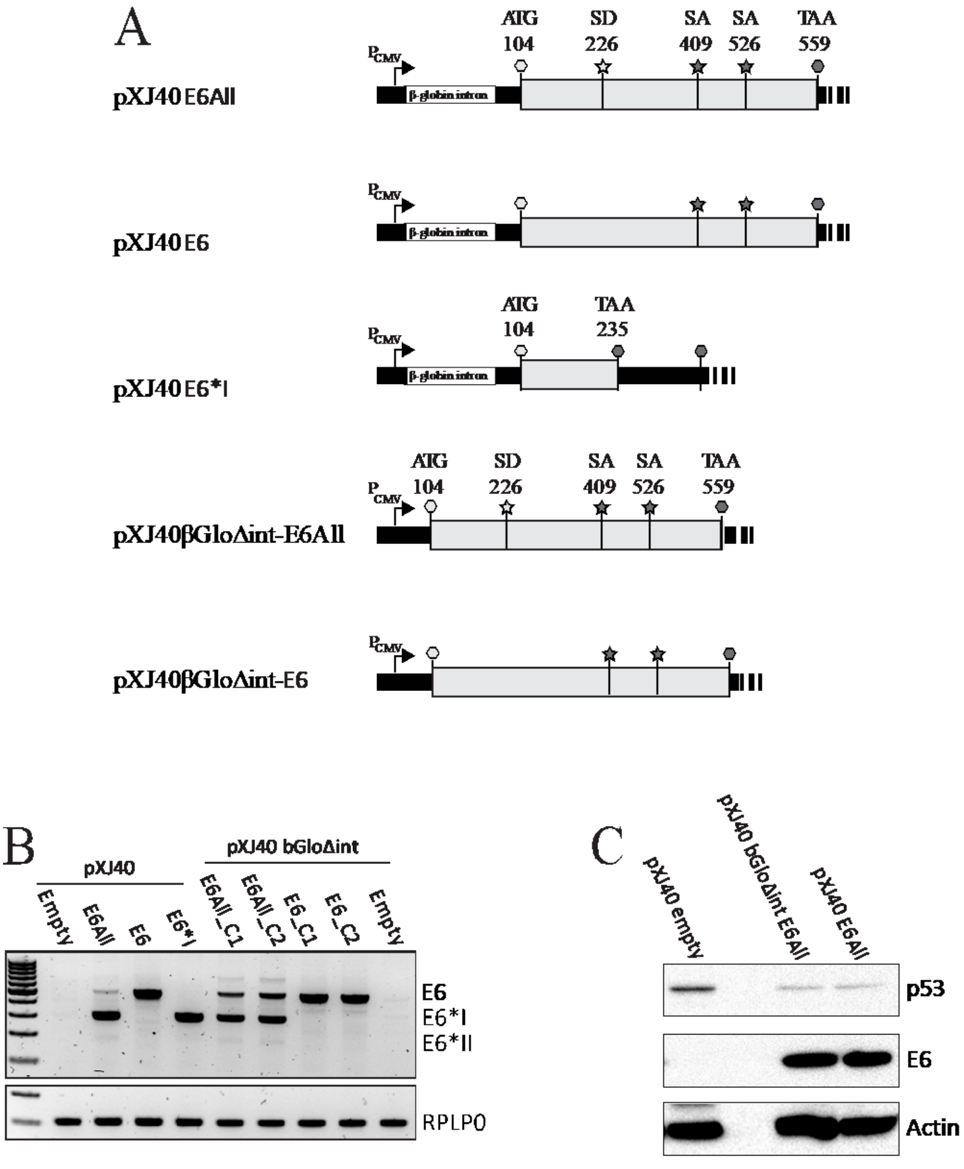

Figure S2. HPV16 E6 and E6*I sequences cloned into pXJ40 or pXJ40βGloΔint vectors. (A) Schematic representation of HPV16 E6 and E6*I constructs used in this study. (B) RT-PCR analysis of alternatively spliced E6 transcripts generated from the pXJ40E6All, pXJ40E6, pXJ40E6*I, pXJ40βGloΔintE6All (clones C1 and C2) and pXJ40βGloΔintE6 (clones C1 and C2) vectors after transient transfection in U-2 OS cell line. pXJ40βGloΔint transfections display a less efficient splicing of E6 transcripts but without altering the overall transcription level of E6 orf. RPLP0 was used as loading control. (C) Western blot analysis showing no change of E6 protein expression in U-2 OS cells transiently transfected with the pXJ40-E6All and pXJ40βGloΔint-E6All vectors.

A

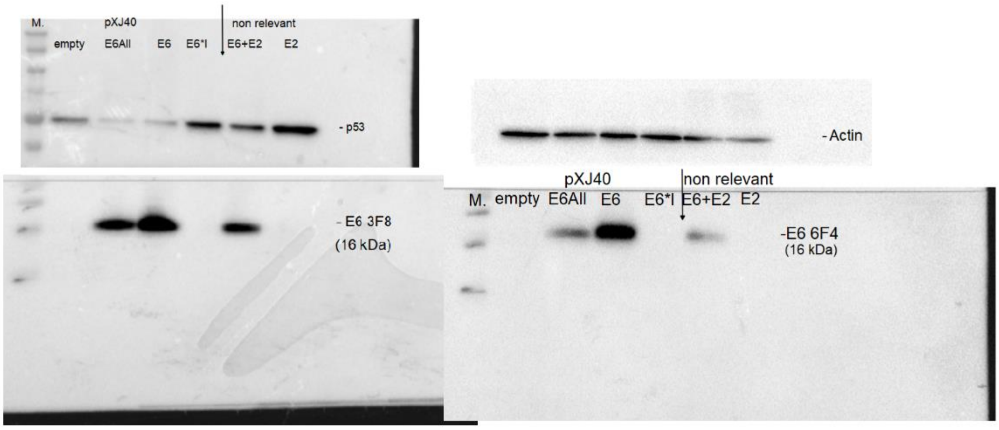

B

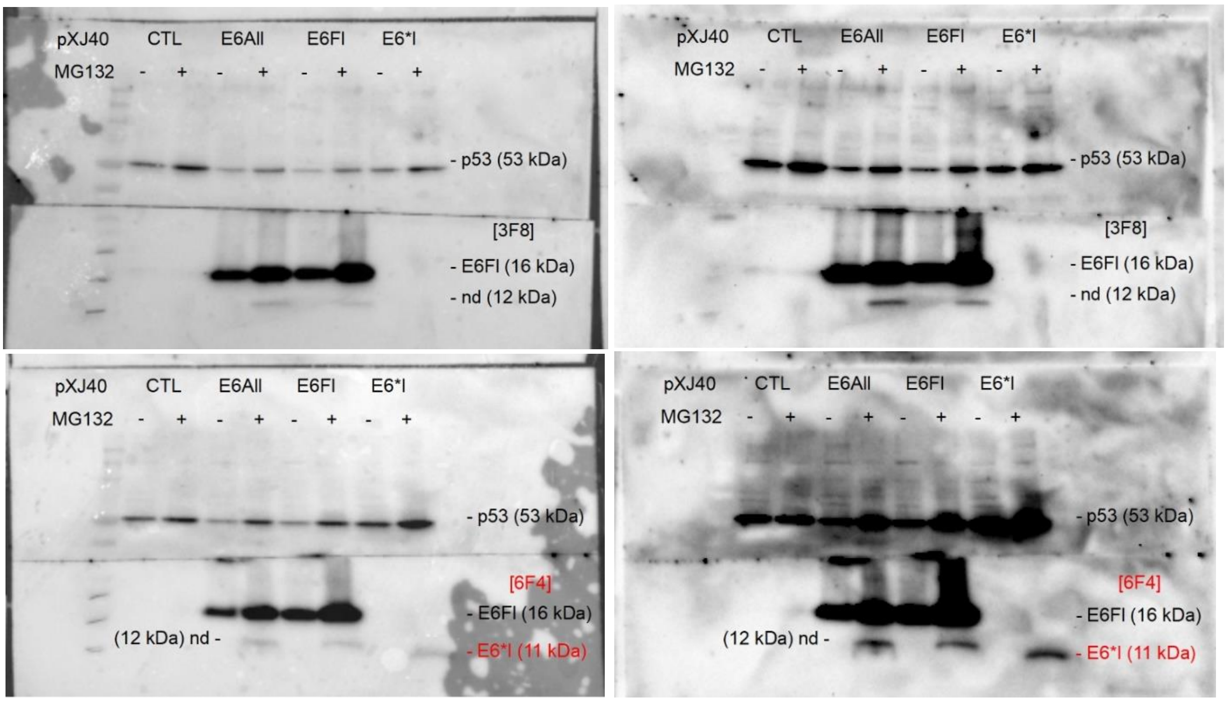

Figure S3. Uncropped Western blot used in figure 1. (A) Figure 1B. E6 (3F8 or 6F4) and p53 hybridizations were performed on the same membrane. (B) Figure 1C. E6 (3F8 or 6F4) and p53 hybridizations were performed on the same membrane. The right panel represent a longer time of exposure compared to the left one. The upper blot of E6 was performed with the 3F8 antibody which does not recognize E6*I, where the lower one was performed with the 6F4 which does [1]. The Precision Plus Protein dual Xtra Standards (Bio-rad) was used as marker for all protein gel migration.

A

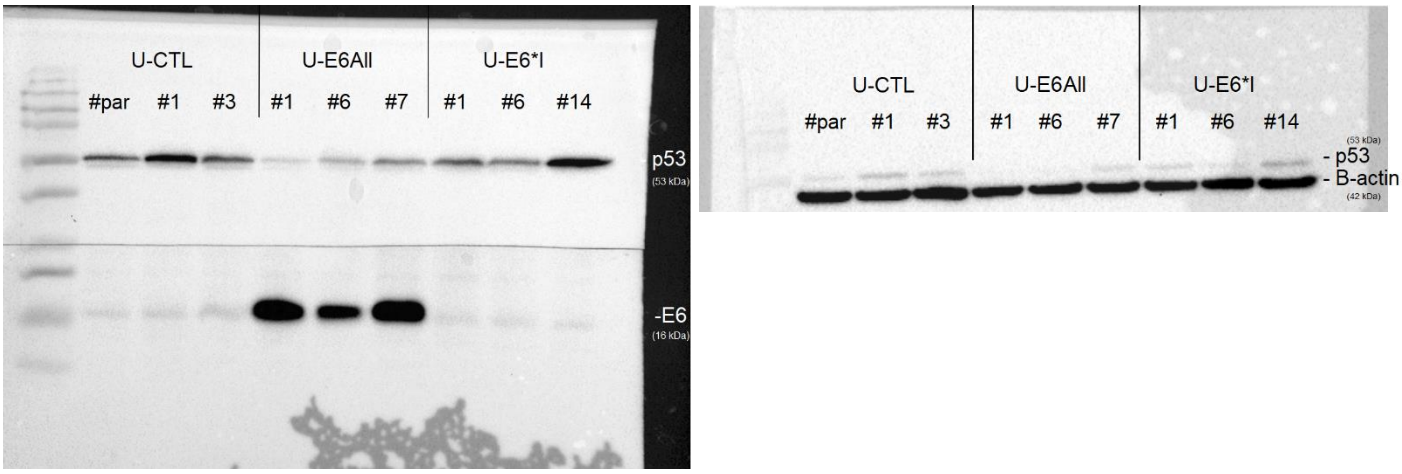

B

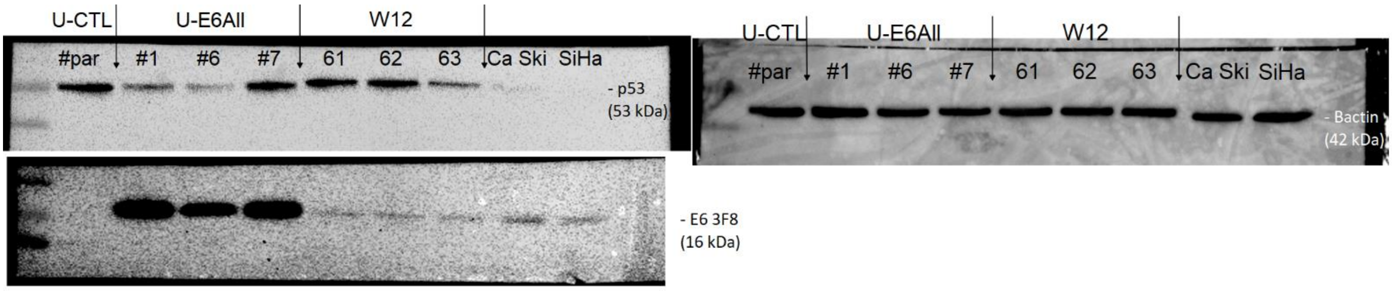

Figure S4. Uncropped Western blot used in figure 2. (A) Figure 2A. B-actin antibody [2] hybridization and revelation were performed after p53 [3] and E6 (3F8), on the same membrane. (B) Figure 2B. All revelations were also done on the same membrane but with a longer time of exposure for E6, and p53 membrane piece was stripped before actin hybridization.

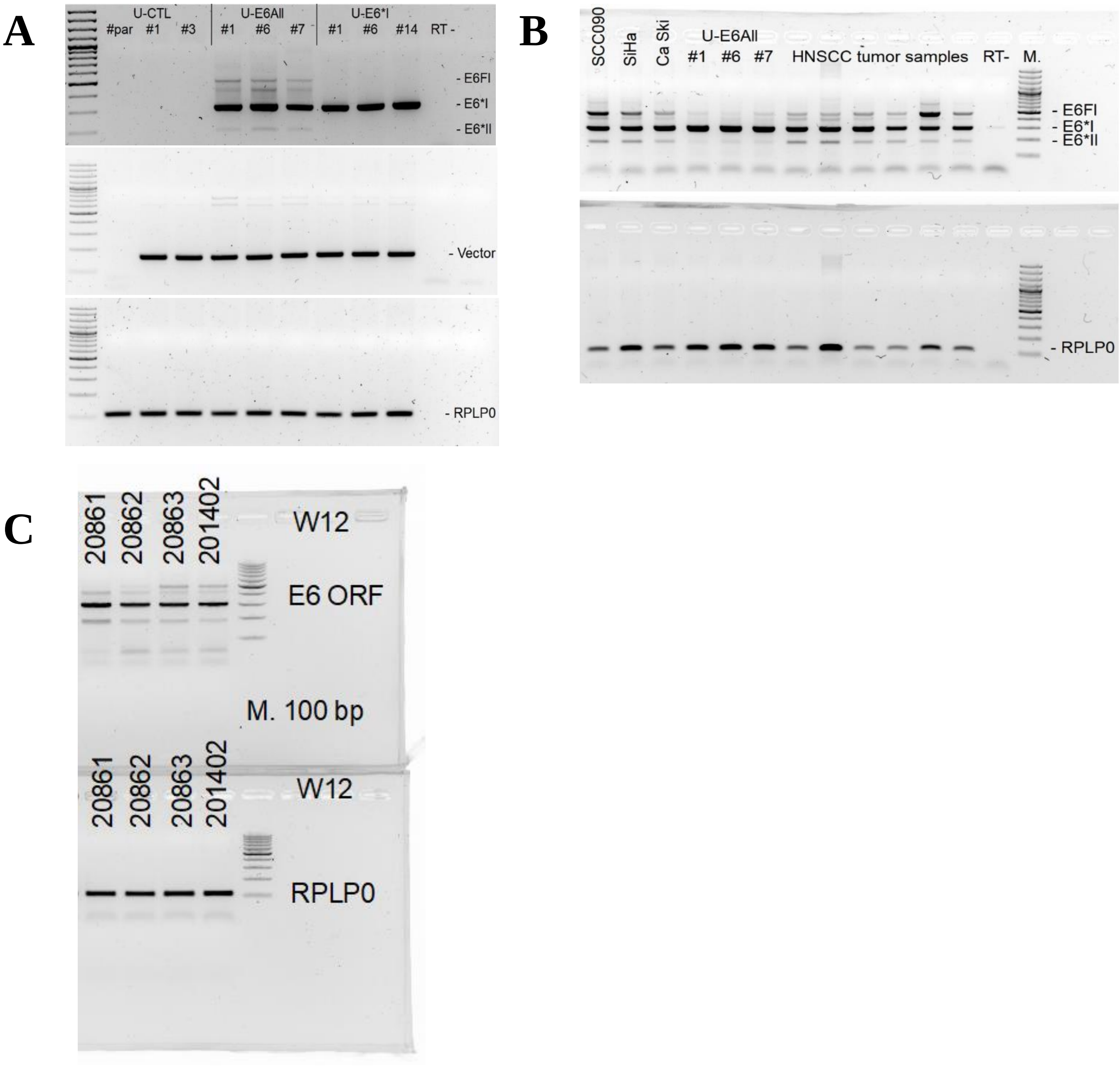

Figure S5. Uncropped RT-PCR Gel used in figure 2. (A) Figure 2C. The GeneRuler DNA ladder 100 bp plus (Thermoscientific) was used as marker for all PCR gel migration. (B) Figure 2D. (C) Figure 2E.

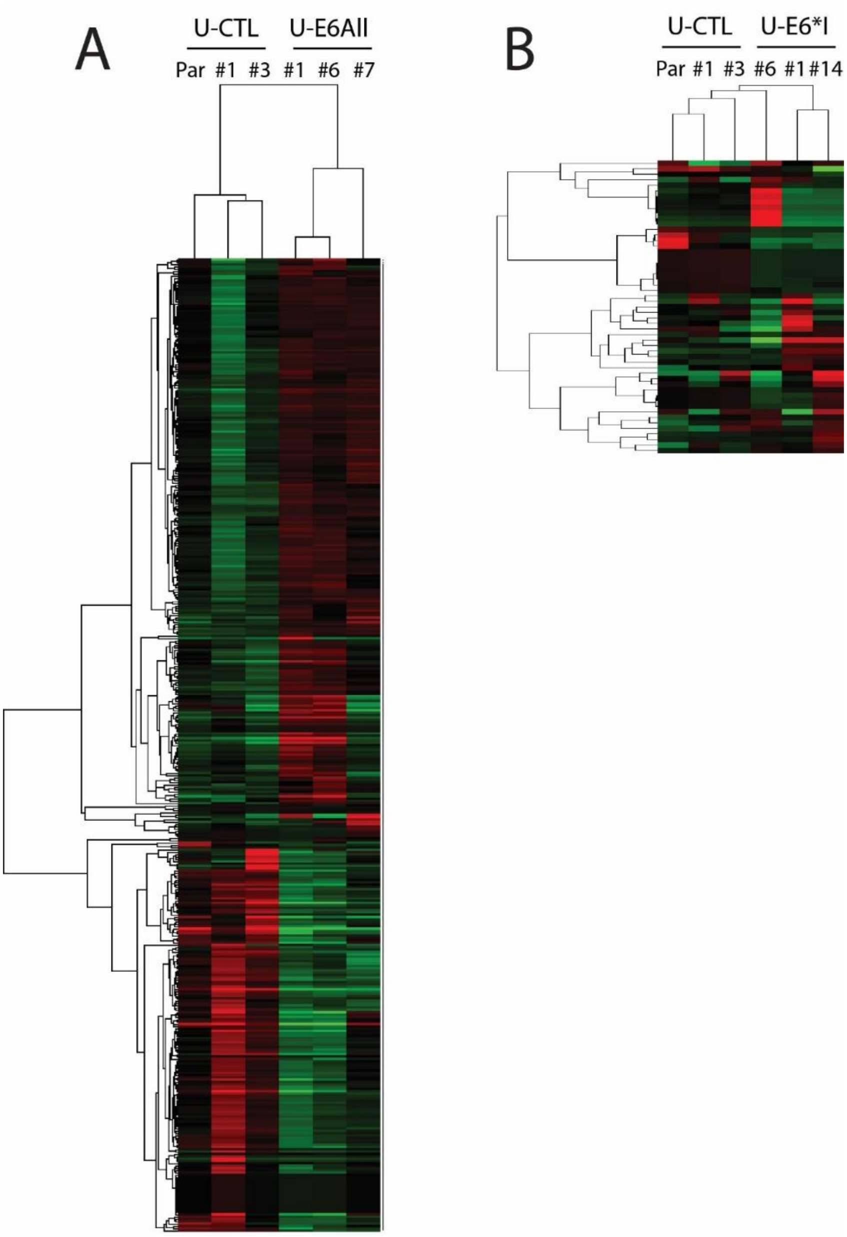

Figure S6. Heatmaps showing gene expression signature for U-E6All vs. U-CTL and U-E6*I vs. U-CTL. (A) The heatmap presentation of the 419 genes significantly deregulated in U-E6All cell lines compared to U-CTL. High (red) and low (green) expression, corresponding to a +7.6 to -5.8 log2 scale of fold change relative to the average of each transcript across all 6 clones. (B) The heatmap presentation of the 53 genes significantly deregulated in U-E6*I cell lines compared to U-CTL. High (red) and low (green) expression, corresponding to a +6.2 to -2.8 log2 scale of fold change relative to the average of each transcript across all 6 clones.

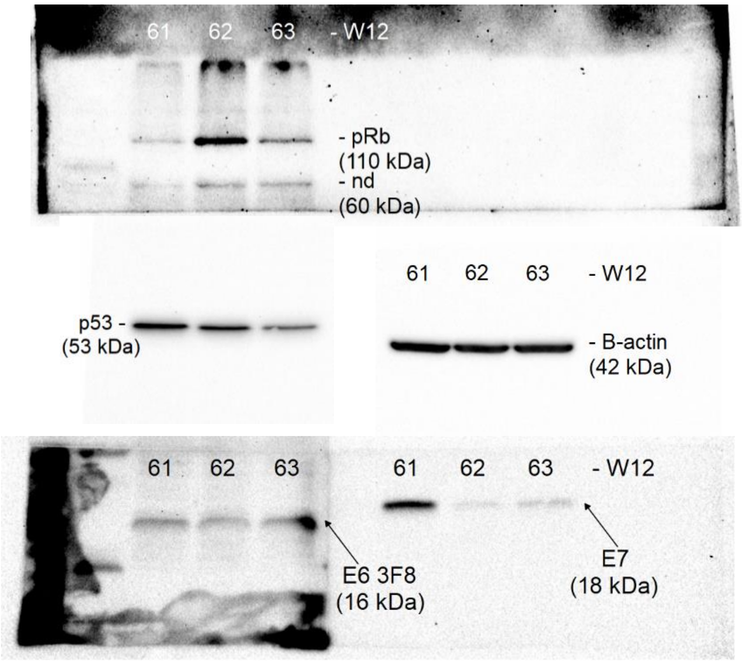

Figure S7. Uncropped Western blot used in figure 8B. The three samples were run in duplicate on the same gel and membrane. Hybridizations and revelation of E6, E7 [4], p53, pRb [5] and B-actin were done on the same membrane.

Supplemental references

1. Lagrange, M. *et al.* Binding of human papillomavirus 16 E6 to p53 and E6AP is impaired by monoclonal antibodies directed against the second zinc-binding domain of E6. *J. Gen. Virol.* **86,** 1001–1007 (2005).

2. Li, M., Brooks, C. L., Kon, N. & Gu, W. A Dynamic Role of HAUSP in the p53-Mdm2 Pathway. *Mol. Cell* **13,** 879–886 (2004).

3. Kojima, Y. *et al.* Adenovirus-mediated transfer of HPV 16 E6/E7 antisense RNA combined with cisplatin inhibits cellular growth and induces apoptosis in HPV-positive head and neck cancer cells. *Cancer Gene Ther.* **25,** 274 (2018).

4. Hoppe-Seyler, K. *et al.* Viral E6/E7 oncogene and cellular hexokinase 2 expression in HPV-positive cancer cell lines. *Oncotarget* **8,** 106342–106351 (2017).

5. Guo, C. *et al.* Potent Anti-Tumor Effect Generated by a Novel Human Papillomavirus (HPV) Antagonist Peptide Reactivating the pRb/E2F Pathway. *PLOS ONE* **6,** e17734 (2011).
